# FtH-Mediated ROS Dysregulation Promotes CXCL12/CXCR4 Axis Activation and EMT-Like Trans-Differentiation in Erythroleukemia K562 Cells

**DOI:** 10.3389/fonc.2020.00698

**Published:** 2020-05-05

**Authors:** Roberta Chirillo, Ilenia Aversa, Anna Di Vito, Alessandro Salatino, Anna Martina Battaglia, Alessandro Sacco, Maddalena Adriana Di Sanzo, Maria Concetta Faniello, Barbara Quaresima, Camillo Palmieri, Flavia Biamonte, Francesco Costanzo

**Affiliations:** ^1^Department of Experimental and Clinical Medicine, “Magna Græcia” University of Catanzaro, Catanzaro, Italy; ^2^Department of Experimental and Clinical Medicine, Research Center of Biochemistry and Advanced Molecular Biology, “Magna Græcia” University of Catanzaro, Catanzaro, Italy; ^3^Interdepartmental Center of Services (CIS), “Magna Græcia” University of Catanzaro, Catanzaro, Italy

**Keywords:** ferritin heavy chain, ROS, CXCR4, EMT, NF-κB, leukemia, tumor microenvironment, hematological malignancies

## Abstract

The cell-microenvironment communication is essential for homing of hematopoietic stem cells in stromal niches. Recent evidences support the involvement of epithelial-to-mesenchymal (EMT) process in hematopoietic stem cell homeostasis as well as in leukemia cells invasiveness and migration capability. Here, we demonstrate that the alteration of iron homeostasis and the consequent increase of redox metabolism, mediated by the stable knock down of ferritin heavy chain (FtH), enhances the expression of CXCR4 in K562 erythroleukemia cells, thus promoting CXCL12-mediated motility. Indeed, addition of the CXCR4 receptor antagonist AMD3100 reverts this effect. Upon FtH knock down K562 cells also acquire an “EMT-like” phenotype, characterized by the increase of *Snail, Slug* and Vimentin with the parallel loss of E-cadherin. By using fibronectin as substrate, the cell adhesion assay further shows a reduction of cell adhesion capability in FtH-silenced K562 cells. Accordingly, confocal microscopy shows that adherent K562 control cells display a variety of protrusions while FtH-silenced K562 cells remain roundish. These phenomena are largely due to the reactive oxygen species (ROS)-mediated up-regulation of HIF-1α/CXCR4 axis which, in turn, promotes the activation of NF-κB and the enhancement of EMT features. These data are confirmed by treatments with either N-acetylcysteine (NAC) or AMD3100 or NF-κB inhibitor IκB-alpha which revert the FtH-silenced K562 invasive phenotype. Overall, our findings demonstrate the existence of a direct relationship among iron metabolism, redox homeostasis and EMT in the hematological malignancies. The effects of FtH dysregulation on CXCR4/CXCL12-mediated K562 cell motility extend the meaning of iron homeostasis in the leukemia cell microenvironment.

## Introduction

The tumor microenvironment (TME) is a major player in cancer progression and several signals, such as oxygen supply, cytokines, and chemokines drive the communication between TME and tumor cells (1–3). The CXCL12/CXCR4 axis promotes tumor cell growth and propagation of distant metastases through the activation of epithelial-to-mesenchymal transition (EMT) (4). During EMT, cancer cells acquire features of mesenchymal-like cells including enhanced migratory and invasive abilities, changes in cellular adhesion and remodeling of the extracellular matrix (5, 6). Cancer cells expressing CXCR4 tend to home to secondary organs where its ligand CXCL12 is actively secreted, mainly by mesenchymal stromal cells (7).

Tumors rapidly exhaust the local oxygen supply creating a hypoxic environment which, in turn, promotes the overproduction of reactive oxygen species (ROS) (8). ROS can induce EMT, but the specificity of their action in the regulation of given EMT markers is dependent on the cellular context and the type of tissue (9–12). Numerous studies have explored the role of ROS in inducing both cell migration and EMT in solid cancer (13, 14). In this regard, we and others have previously reported that the knock down of ferritin heavy chain (FtH), the catalytic subunit of the human ferritin, promotes cell motility through either the activation of CXCR4 signaling or the induction of EMT in a variety of solid cancer *in vitro* models including breast and lung cancer cell lines (15–17).

The trafficking of tumor cells represents a key process that contributes to progression also of hematological malignancies such as myeloid and lymphoid leukemias or multiple myeloma (18, 19). A common feature of these tumors is the homing and infiltration of hematological cancer cells into the bone marrow (BM) which supports initiation, maintenance and proliferation of the malignant cells (7). Both homing and migration of leukemic stem cells are regulated by niche cells living in the BM through the activation of the CXCL12/CXCR4 axis signaling (20–22). Indeed, blocking CXCL12 binding to CXCR4 with the specific CXCR4 inhibitor AMD3100 disrupts hematological neoplastic cells interaction with the BM microenvironment (21).

In chronic myelogenous leukemia (CML) cells, CXCR4 activates PI3K/AKT signaling pathway and promotes the translocation of NF-κB complexes into nucleus thereby decreasing the expression of pro-apoptotic proteins (23, 24). Moreover, CXCL12 activates pro-survival signal pathways including those mediated by MAPK, S-6-kinase, STAT3 and STAT5, and *in vitro* treatment with CXCR4 antagonists inhibits cell growth and induces cell death (25, 26). The molecular mechanisms regulating the expression of CXCR4 in hematological malignancies have therefore been largely investigated. Numerous evidences show that hypoxia in BM leads to increased HIF-1α transcriptional activity on CXCR4 expression resulting in enhanced migration and homing of circulating malignant cells to new BM niches (27–29).

During the last decade, EMT has gained increasing attention also in hematological malignancies. Few reports indicate that EMT-transcription factors (TFs), including Twist-1 and Slug, are implicated in hematopoietic stem cell self-renewal by interacting with stemness signaling key factors c-Myc and c-Kit (30, 31) while Slug up-regulation promotes leukemogenesis and confers resistance to apoptosis in leukemia cells (32). In addition, imatinib-resistant CML cells exhibit a so-called “EMT-like” phenotype along with increased invasion and migration properties both *in vitro* and *in vivo* (33). Overall these data suggest that EMT might play significant role in inducing tumor dissemination and thus chemoresistance also in hematological malignancies; however, this topic still has remarkable gaps to overwhelm.

In this study, we address for the first time the role of FtH-induced ROS increase in bestowing mesenchymal properties to hematological cells. To achieve this goal, we defined the effects of FtH knock down in the induction of EMT markers, activation of CXCR4/CXCL12 signaling pathway and migration of K562 erythroleukemia cells, and further attempted to understand the molecular mechanisms involved.

## Materials and Methods

### Cell Culture and Treatment

K562, a human erythroleukemia cell line (ATCC number CCL-243), was cultured as described in Di Sanzo et al. (34). The human stromal cells HS5, were cultured in DMEM medium supplemented with 10% fetal bovine serum and antibiotics at 37°C in an atmosphere of humidified air containing 5% CO_2_. Lentiviral preparations and transductions were performed as previously described using a shRNA as control (K562^shRNA^) or a shRNA that targets the 196–210 region of the *FtH* mRNA (K562^shFtH^) (35). All the experiments were performed using a puromycin-selected pool of clones (1 μg/mL) (Sigma Aldrich, St. Louis, MI, USA). K562 cells were transfected using the Nucleofector system from Amaxa (Lonza, Basel, Switzerland) according to the manufacturer's optimized protocol. To evaluate the role of NF-κB in inducing EMT-like features, we over-expressed the NF-κB inhibitor IκB-α using a homemade pRc/CMV-HA-IκB-α plasmid and its empty control kindly provided by Prof. Ileana Quinto (Magna Graecia University of Catanzaro, Italy) as previously described by Aversa et al. (36). CXCL12 was added to K562 cell culture medium at a final concentration of 100 ng/ml. N-acetylcysteine (NAC) was added to the K562 cell culture medium at a final concentration of 5 mM for 2 h. Plerixafor (AMD3100) was added to the K562 cell culture medium at a final concentration of 10 μM for 1 h.

### Protein Extractions

Protein extractions were performed on K562^shRNA^, K562^shFtH^, K562^shFtH/pRc/CMV^, K562^shFtH/pRc/CMV−3HA−Iκ*B*^ and NAC treated cells. Briefly, for total protein extractions, K562 cells were lysed in ice-cold radioimmunoprecipitation assay (RIPA) buffer containing protease inhibitors as described by Zolea et al. (37). For the quantification of nuclear p65 amounts, protein extraction from nucleus was performed as previously described by Aversa et al. (36).

### Western Blotting Analysis

A total of 40 μg protein extract was boiled for 10 min in SDS sample buffer, separated by 12% SDS-PAGE and transferred to a nitrocellulose membrane by electroblotting as reported in Di Sanzo et al. (38). The nitrocellulose membranes were incubated overnight at 4°C with the following antibodies: (a) anti-CXCR4 (1:500; Abcam), (b) anti-HIF-1α (H-206) (1:200; Santa Cruz Biotechnology), (c) anti-p65 (C-20) (sc-372, 1:1,000; Santa Cruz Biotechnology), (d) anti-HDAC (1:5,000; Sigma-Aldrich), (e) anti-HA probe (F-7) (1:1,000; Santa Cruz Biotechnology), (f) anti-Vimentin, (g) anti-E-cadherin, (h) anti-Snail, (i) anti-Slug (1:1,000; Cell Signaling Technology, Danvers, MA, USA), (l) anti-FtH (1:200; Santa Cruz Biotechnology), (m) anti-γ-Tubulin (C-20) (1:2,000; Santa Cruz Biotechnology), (n) anti-Nucleolin (D4C7O) (1:1,000; Cell Signaling Technology) over-night at 4°C, followed by incubation with goat anti-rabbit and mouse anti-goat secondary antibodies (1:5,000; Santa Cruz Biotechnology). Membranes were incubated with horseradish peroxidase (HRP)-conjugated secondary antibodies and immunoreactive bands were visualized with the ECL Western blotting detection system (BioRad, Hercules, CA, USA).

### Quantification of CXCR4 Surface Expression

K562 cells (2 × 10^5^) were harvested and rinsed once. Then, the cells were incubated with anti-CXCR4 antibody (1:400; Abcam) for 1 h at 4°C. After primary antibody incubation, the cells were rinsed with 1X PBS and incubated for 30 min with Alexa Fluor 633 donkey anti-goat antibody (H+L) (1:400) resuspended in 5 mg/ml BSA and 0.76 mg/ml EDTA. The cells were rinsed with 1X PBS, resuspended in 300 μl 1X PBS and evaluated by a FACS BD LSRFortessa^TM^ X-20 cytofluorometer (BD Biosciences, San Jose, CA, USA).

### Immunofluorescence

K562 cells were cultured on cover slip coated with fibronectin for 24 h. Samples preparation was performed as reported in Biamonte et al. (39). Thereafter, these cover slips were incubated for 1 h with primary antibodies anti-Vimentin (clone V9, ready to use, Dako) and 1 h with secondary antibody FITC-conjugated anti-mouse diluted in blocking buffer. For E-cadherin staining, the cover slips were incubated overnight at 4°C with primary antibody (clone 24E10, 1:200, Cell Signaling) in a humidified room, and for 1 h with Alexa Fluor 488-conjugated anti-rabbit, both diluted in blocking buffer. To stain actin filaments, cells were incubated for 30 min in this buffer containing Alexa Fluor 488 phalloidin at 1:40 dilution (Thermo Fisher Scientific, Waltham, Massachusetts, USA). After 3 washes with PBS, nuclear DAPI (1:500, Invitrogen, Carlsbad, CA) was added for 20 min. The samples were mounted on microscope slides using a mounting solution ProLong Gold antifade reagent (Thermo Fisher Scientific). Images were collected using a Leica DM-IRB/TC-SP2 confocal microscopy system (63× objective).

### Cell Adhesion Assay

K562^shRNA^, K562^shFtH^ and K562^shFtH^ NAC-treated cells were subjected to adhesion assays using Fibronectin as an adhesion substrate. Briefly, 6-well flat-bottom plates were incubated overnight at 37°C with 5 μg/cm^2^ of Fibronectin in PBS. After gentle washing with PBS and incubation with 1% BSA for 1 h at room temperature, cells (3 × 10^4^ cells/well) were added and allowed to adhere for 24 h at 37°C. Non-adherent cells were then removed by washing each well with PBS and adherent cells were counted using the cell count function in Image J 1.42 software, on ten fields per well. Each field consisted of a photo obtained at 200× magnification.

### Migration Assay

K562^shRNA^, K562^shFtH^ and K562^shFtH^ NAC-treated cells were used to the migration assay using as chemoattractant CXCL12 chemokine as already described by Aversa et al. (15). After 18 h of incubation, the upper chambers were removed, and the cells in the lower chambers were counted using an optical microscope. Migration assay using the conditioned media derived from stromal cells HS5 (HS5-CM) was assessed in 24-well plate and polycarbonate filters with an 8.0 μm pore size. First, the supernatant media of HS5 cells was collected after 12 h and passed through a 0.45 μm filter. Briefly, 2 × 10^5^ K562 cells were harvested, suspended in 200 μl serum-free RPMI with 1% BSA and placed in the upper chamber. The lower chambers contained 500 μl of HS5-CM. The plates were incubated at 37°C in 5 % CO2 for 8 h. The upper chambers were removed, and the cells in the lower chambers were counted using an optical microscope. The cell migration is expressed as the percentage of increase compared with the corresponding control.

### ROS Detection

ROS were determined by incubating 2 × 10^5^ K562^shRNA^, K562^shFtH^ and K562^shFtH^ cells NAC-treated with 1μM redox-sensitive probe 2′-7′-DCF (CM-H2CFDA; MolecularProbes, Eugene, OR, USA) for 30 min at 37°C. Afterward, pellet was washed twice with 1X PBS, then the pellet was resuspended in 1X PBS and analyzed using a FACS BD LSRFortessa^TM^ X-20 cytofluorometer (BD Biosciences).

### RNA Isolation and qPCR Analysis

Total RNA isolation was performed as previously reported in Sottile et al. (40). Gene expression analysis was assessed by real-time PCR using the cDNA obtained from K562^shRNA^, K562^shFtH^, K562^shFtH/pRc/CMV^, K562^shFtH/pRc/CMV−3HA−Iκ*B*^ cells and K562^shFtH^ cells treated with NAC.

Real time PCR was performed ad reported in Biamonte et al. (41). Briefly, 50 ng of cDNA was amplified in 20 μl of reaction mix containing Power SYBR Green PCR Master mix (Thermo Fisher Scientific) and the expression of *CXCR4, FtH, E-cadherin, HIF-1*α, *Snail, Slug* and *Vimentin* were analyzed. The human *GAPDH* cDNA fragment was amplified as the internal control. Data analysis was performed using the 2^−ΔΔCt^.

### Statistical Analysis

All experiments were conducted at least three times, and the results are reported as mean values ± standard deviations (SD). Data analysis was performed by Student's-*t*-test assuming equal variances. *p*-values ≤ 0.05 were considered statistically significant.

## Results

### *FtH* Knock Down Promotes CXCL12/CXCR4 Axis Activation and Motility in K562 Cells

**Figure 1:**
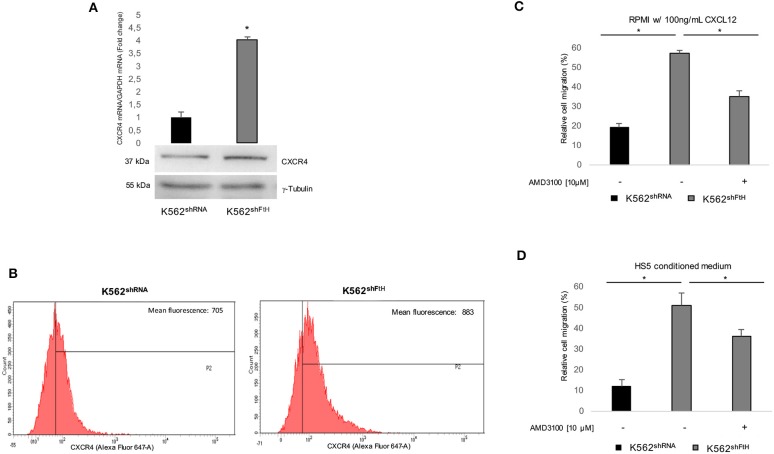
CXCR4/CXCL12 axis is activated in K562^shFtH^ cells. **(A)** qPCR and western blot analysis of CXCR4 in K562^shRNA^ and K562^shFtH^ cells. Final results represent mean ± SD of three independent experiments, ^*^p < 0.05. γ-Tubulin was used as loading control in WB experiments **(B)** Flow cytometry analysis of CXCR4 in K562^shRNA^ and K562^shFtH^ cells. Data are representative of three independent experiments **(C)** CXCL12 dependent-cell migration was examined in 24-well plates. Cells were placed in the upper chamber (8 μm) in nude RPMI. After 8 h, cells migrated toward medium containing CXCL12 (100 ng/ml) were counted using an optical microscope. Data are reported percentage of migrated cells. Final results represent mean ± SD of three independent experiments, ^*^p < 0.05. **(D)** Migration analysis of K562^shRNA^ and K562^shFtH^ cells performed in 24-well plates. Cells were allowed to migrate for 8 h into the lower chamber containing conditioned medium of HS5 cells. Migrated cells were counted, and the percentage of migrated cells was calculated. Final results represent mean ± SD of three independent experiments, ^*^p < 0.05.

Earlier studies by others and us indicate that FtH interacts with internalized olo-CXCR4 receptor thereby suppressing the downstream signaling pathway in a variety of epithelial tumor cells (15, 16). As experimental model we used a pool of erythroleukemia K562 cell clones stably silenced for *FtH* (K562^shFtH^). FtH mRNA and protein expression levels in K562^shFtH^ and in K562^shRNA^ control cells are reported in Figure S1A. To assess the role of FtH on CXCR4 signaling in hematological tumors, we first measured CXCR4 levels in K562^shFtH^ and in K562^shRNA^ and we found that *FtH* knock down significantly enhanced CXCR4 expression at both mRNA and protein levels (Figure 1A). Flow cytometry analysis revealed an increase of about 25% in CXCR4 cell surface expression in K562^shFtH^ cells compared to K562^shRNA^ control cells (Figure 1B). Next, to determine whether the CXCR4 increase modulates the cell migration ability, we cultured both K562^shRNA^ and K562^shFtH^ cells either in RPMI medium supplemented with 100 ng/ml CXCL12 or in a HS5 mesenchymal stromal cells-conditioned medium for 8 h. As controls, we prepared parallel cultures of K562 cells with RPMI complete medium alone (data not shown). Cell migration assays demonstrate that K562^shFtH^ cells exhibit a higher migration ability of about 5-fold compared to K562 control cells upon exposure to both CXCL12 or HS5-conditioned medium. Accordingly, treatment with the specific CXCR4 inhibitor AMD3100 (10 μM for 8 h) significantly decreased the migration of K562^shFtH^ in both modified culture media (Figures 1C,D).

### *FtH* Silencing Induces Mesenchymal-Like Features in K562 Cells

**Figure 2:**
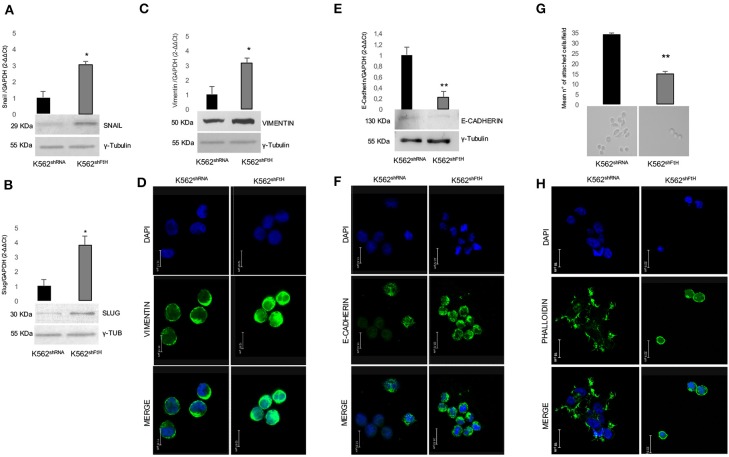
Analysis of EMT markers in K562 cells upon FtH silencing. qPCR and Western Blot analysis of **(A)** Snail, **(B)** Slug, **(C)** Vimentin and **(E)** E-Cadherin in K562^shRNA^ and K562^shFtH^ cells. Final results are reported as mean ± SD of three independent experiments, *p < 0.05. γ-Tubulin was used as loading control in WB experiments. Immunofluorescence staining for **(D)** Vimentin and **(F)** E-Cadherin in K562^shRNA^ and K562^shFtH^ cells. Images were collected using a Leica TCS SP2 confocal microscopy system (63X). Data are representative of three independent experiments **(E)**. **(G)** Cell adhesion assay in K562^shRNA^ and K562^shFtH^ cells performed in a 6-well plate coated with 5 μg/mL fibronectin. Results are reported as the mean number of adherent cells counted per field upon 24 h through the optical microscope, **p < 0.01 (upper panel). A representative image of adherent K562^shRNA^ and K562^shFtH^ cells obtained from optical microscope (lower panel). **(H)** Representative images of immunofluorescence staining for Phalloidin in K562^shRNA^ and K562^shFtH^ cells. Images were collected using a Leica TCS SP2 confocal microscopy system (63X).

We then explored the effect of *FtH* knock down on the classic EMT markers E-cadherin and Vimentin, as well as on the two EMT-transcription factors (EMT-Tfs) Snail and Slug. Real-time PCR and WB analyses clearly indicate a consistent increase in the steady-state amounts of both EMT-Tfs in K562^shFtH^ cells compared to K562^shRNA^ cells (Figures 2A,B). Accordingly, upon *FtH* silencing, the expression of Vimentin appears roughly triplicated in parallel with a significant decrease of E-cadherin amounts either at mRNA or protein level (Figures 2C–F).

Besides increased motility, reduced cell-cell adhesion capability is a key feature of the EMT process. By using fibronectin as substrate, we analyzed the adhesion ability of K562^shFtH^ and control cells, founding that the FtH-silenced cells halved their capability to adhere to the substrate (Figure 2G, upper panel). Optical imaging of K562^shRNA^ cells reveals that they behave as adherent cells stucking to fibronectin through cell protrusion, while the fraction of adherent K562^shFtH^ remains roundish (Figure 2G, lower panel). Confocal microscopy shows F-actin aggregates, cell-surface protrusions and/or extensions with a complex network of actin filaments (pseudopodia, lamellipodia) and actin bundles (filopodia) in K562^shRNA^ cells. In K562^shFtH^ cells, instead, F-actin is organized in a three-dimensional network beneath the plasma membrane, which likely accounts for the rounded shape of these cells (Figure 2H).

### ROS Increase Induces CXCR4 Signaling and EMT *Trans*-Differentiation Process in K562^shFtH^ Cells

**Figure 3:**
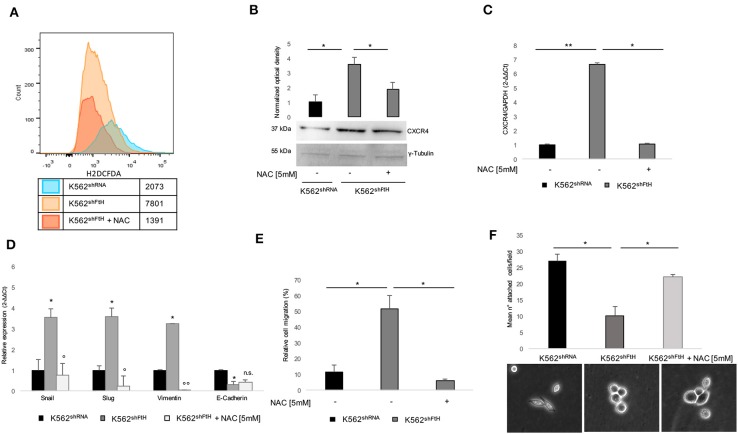
Effects of NAC treatment on EMT transdifferentiation in K562^shFtH^ cells. **(A)** Flow cytometry analysis of ROS in K562^shRNA^ and K562^shFtH^ cells treated and not treated with 5 mM NAC for 2 h. Cells were labeled with H2DCFDA and the assay was performed in triplicate. **(B)** Western blot analysis (lower panel) and optical densitometry (upper panel) of CXCR4 in K562^shRNA^ and K562^shFtH^ cells treated and not treated with 5 mM NAC. Final results are reported as mean ± SD of three independent experiments, *p < 0.05. γ-Tubulin was used as loading control. **(C)** qPCR analysis of *CXCR4* in K562^shRNA^ and K562^shFtH^ cells treated and not treated with 5 mM NAC. Final results represent mean ± SD of three independent experiments, *p < 0.05; **p < 0.01. **(D)** qPCR analysis of *Snail, Slug, Vimentin*, and *E-Cadherin* mRNA expression in K562^shRNA^, K562^shFtH^ and K562^shFtH^ cells treated with NAC. Final results represent mean ± SD of three independent experiments, *p < 0.05 compared to K562^shRNA^ cells. °*p* < 0.05, ^°°^*p* < 0.01 compared to K562^shFtH^ cells. n.s., not significant. **(E)** Migration analysis in K562^shRNA^ and K562^shFtH^ cells treated and not treated with NAC was examined in 24-well plates. After 8 h, cells migrated toward CXCL12 (100 ng/ml) were counted using an optical microscope. Data are reported as the percentage of migrated cells. Final results represent mean ± SD of three independent experiments, *p < 0.05. **(F)** Cell adhesion assay in K562^shRNA^, K562^shFtH^ and K562^shFtH^ cells treated with NAC were cultured in a 6-well plate coated with 5 μg/mL fibronectin. After 24 h adherent cells were counted using an optical microscope; data are reported as mean of the number of adherent cells per field (upper panel), *p < 0.05. A representative image of adherent K562^shRNA^, K562^shFtH^ and K562^shFtH^ cells treated with NAC obtained from optical microscope (lower panel).

We have already reported that *FtH*-silencing induces, in a variety of cell types including K562, a dysregulation of redox homeostasis ending in a consistent ROS overproduction (15, 36, 42, 43). Given the role of ROS in mediating the communication between tumor cell and tumor microenvironment (TME), we sought to explore the effects of the antioxidant agent NAC on both CXCR4 activation and EMT trans-differentiation process of K562^shFtH^. To this, we first re-determined the intracellular levels of ROS by using the DCF-DA assay. As shown in Figure 3A, a 4 h treatment with 5 mM NAC strongly reduced the amounts of ROS in the silenced cells. The decrease of ROS is accompanied by the reversal of the majority of the phenomena induced by FtH-silencing; indeed, the intracellular protein amount of CXCR4 is consistently reduced (Figure 3B) as well as its messenger RNA (Figure 3C) while its cell surface expression is only slightly affected (data not shown). Accordingly, EMT markers expression is down-regulated, with the exception of *E-cadherin* (Figure 3D), and the migratory ability is also consistently impaired (Figure 3E). The percentage of K562^shFtH^ cells adherent to fibronectin substrate is increased (Figure 3F, upper panel), together with the lack of roundish morphology and with a partial recovery of cell protrusions (Figure 3F, lower panel).

### ROS Orchestrate EMT *Trans*-Differentiation Process by Acting on HIF-1α/CXCR4/NF-κB Axis in K562^shFtH^ Cells

**Figure 4:**
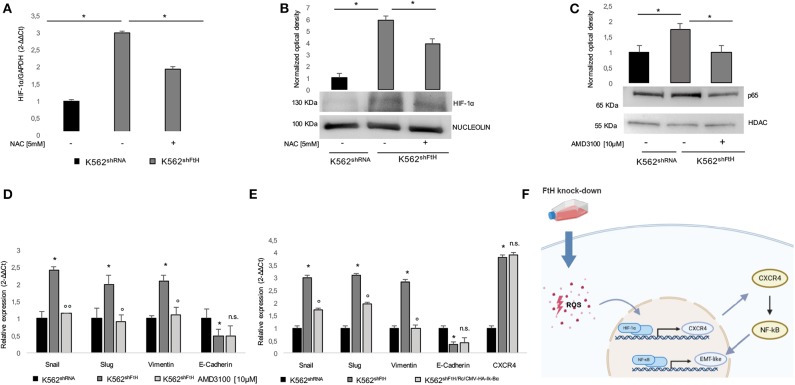
ROS/ HIF-1α/ CXCR4/ NF-kB axis activates EMT in K562^shFtH^ cells. **(A)** qPCR analysis of *HIF-1*α in K562^shRNA^ and K562^shFtH^ cells treated and not treated with NAC. Final results represent mean ± SD of three independent experiments, *p < 0.05. **(B)** Western Blot analysis of HIF-1α (lower panel) and optical densitometry (upper panel) in K562^shRNA^ and K562^shFtH^ cells treated and not treated with NAC. Final results are reported as mean ± SD of three independent experiments, *p < 0.05. γ-Tubulin was used as loading control. **(C)** Western Blot analysis of p65 (lower panel) and optical densitometry (upper panel) in K562^shRNA^ and K562^shFtH^ cells treated and not treated with AMD3100. Final results are reported as mean ± SD of three independent experiments, *p < 0.05. HDAC was used as loading control of nuclear protein fraction. **(D)** qPCR analysis of *Snail, Slug, Vimentin*, and *E-Cadherin* mRNA expression in K562^shRNA^ and K562^shFtH^ cells treated and not treated with AMD3100. Final results represent mean ± SD of three independent experiments, *p < 0.05, compared to K562^shRNA^ cells. °*p* < 0.05, ^°°^*p* < 0.01, compared to KK562^shFtH^ cells. n.s., not significant. **(E)** qPCR analysis of *Snail, Slug, Vimentin, E-Cadherin*, and *CXCR4* mRNA expression in K562^shRNA^, K562^shFtH^ and K562^shFtH/Rc/CMV−HA−Ik−Bα^ cells. Final results represent mean ± SD of three independent experiments, *p < 0.05, compared to K562^shRNA^ cells. °*p* < 0.05, compared to K562^shFtH^ cells. n.s., not significant. **(F)** Cartoon of ROS/ HIF-1α/ CXCR4/ NF-kB axis activating EMT in K562^shFtH^ cells.

Next, we analyzed HIF-1α expression levels in the *FtH* silenced cells, given its function as transcriptional factor of CXCR4. As shown in Figures 4A,B, *FtH* silencing induced HIF-1α up-regulation at both mRNA and protein levels and this effect was significantly attenuated by treatment with 5 mM NAC for 2 h. It has been recently found that CXCR4 modulates PI3K/Akt/NF-κB signaling pathway and that both NF-κB and CXCR4 belong to a regulatory network driving the migration of cancer stem cells (24). Moreover, NF-κB is currently considered a master regulator of cancer cells aggressive phenotype through the direct transcriptional activation of EMT genes in solid tumors (10, 44, 45), and we recently demonstrated its ROS-mediated activation in K562^shFtH^ cells (36).

Therefore, we assessed the effects of the CXCR4 inhibitor AMD3100 on the phenotype acquired by K562 cells upon *FtH* silencing. As shown in Figures 4C,D, p65 nuclear accumulation in K562^shFtH^ cells was significantly reduced upon AMD3100 treatment and this was accompanied by a consistent decrease of all the EMT markers except for *E-Cadherin* that appeared unaffected. This effect was dependent on NF-κB transcriptional activity since blocking NF-κB with the specific inhibitor Ik-Bα suppressed *Snail, Slug* and *Vimentin* upregulation in K562^shFtH^ cells (Figure 4E). The expression of Ik-Bα in K562^shFtH^ cells is reported in Figure S1B.

These results strongly suggest that FtH is involved in bestowing K562 cells with more migratory and more mesenchymal-like features through the hypoxia-induced activation of CXCR4 and p65 transcriptional activation of selected EMT markers (Figure 4F).

## Discussion

Tumor cell migration is a critical process that contributes to the development and progression of both solid and hematological malignancies (46, 47). In solid tumors, the EMT process enhances the metastatic potential converting polarized epithelial cells into non-polarized mesenchymal cells thus promoting cell mobility, invasion and resistance to apoptotic stimuli (48–50). In hematological malignancies, the blasts move from BM into peripheral blood and colonize distant sites such as liver and spleen, a process reminiscent of EMT in metastatic solid tumors (51–53). Moreover, it has been recently demonstrated that EMT transcription factors are critical in promoting leukemia and lymphoma progression (6, 18).

Many of the molecules driving homing and retention of leukemic cells in tissues have been identified (54, 55); among them, the CXCL12/CXCR4 axis has been shown to be essential for hematopoietic stem cell (HSC) migration and homing and also for cancer cell migration and metastasis (3, 20, 56). In particular, CXCR4 expression is necessary to keep the leukemic cells in the CXCL12-enriched BM microenvironment, and the efficient blockade of CXCR4 mobilizes the cells from the BM into the circulation (3, 19, 22).

In solid cancers, a tumor microenvironment that is rich in reactive oxygen species (ROS) promotes the binding of the hypoxia inducible factor subunit HIF-1α to its response element (HRE) in the promoter region of CXCR4, thus critically influencing CXCR4-mediated expression and functions, and ultimately encouraging cancer metastasis (27, 28). Hypoxia represents a key driver of metabolic reprograming also in the leukemic BM niche where it is often associated with increased production of ROS (27, 57). This feature has been observed in numerous leukemic cell lines and also in cells from patients with CML and AML (58–60). Growing evidences suggest the role of ROS-mediated metabolic alterations in triggering hematopoietic cancer cell mobilization (54, 61).

In the last years, we and others have demonstrated that the knock down of ferritin heavy chain (FtH) induces EMT in epithelial derived cell lines, mainly though not exclusively by increasing ROS production (15, 17). The role of ferritin in hematological malignancies has been explored as well (42, 62, 63). In chronic myelogenous leukemia K562 cells *FtH*-silencing, by altering the redox metabolism, triggers p65 nuclear activation and resistance to doxorubicin (36).

In this study, we demonstrate that *FtH* knock-down promotes a quasi-mesenchymal phenotype and enhances mobility in K562 cells through the activation of a molecular axis arising from ROS mediated-induction of HIF-1α/CXCR4 and ending in p65-mediated transcriptional activation of the mesenchymal markers *Snail, Slug* and *Vimentin*.

In detail, K562 cells react to *FtH* knock down-induced oxidative stress by enhancing the expression of the regulatory HIF-1α subunit that, in turn, acts as a transcription factor for *CXCR4*. According to these results, ROS attenuation with NAC specifically reduced CXCR4 mRNA and protein levels induced by hypoxia. In K562^shFtH^ cells, CXCR4 up-regulation promotes the nuclear translocation of p65 subunit belonging to the transcriptional complex NF-κB. There is increasing evidence suggesting a reciprocal interplay between CXCR4 and NF-κB signaling in fine tuning cancer cellular signaling pathways (23, 24). Although the vast majority of data report that NF-κB contributes to the increase in CXCR4 expression (64), few recent reports suggest the possible existence of a regulatory feedback loop (24). Our results highlight that, besides being redox sensitive, the activation of NF-κB in K562^shFtH^ cells is also dependent on CXCR4 increase, being reversible upon AMD3100 inhibitor treatment.

A positive correlation between NF-κB activation and EMT has been described in several human solid tumors including breast cancer (44), prostate cancer (65), renal carcinoma (66) and head and neck squamous cell carcinomas (67). A number of studies have also recently demonstrated that NF-κB regulates the transcription of EMT-inducing factors *Slug, Twist* and *Sip1* (68). Our results provide further evidence regarding NF-κB's involvement in EMT regulation also in the hematological malignancies since, in K562^shFtH^ cells, p65 nuclear translocation is accompanied by the over expression of the two key EMT-TF *Snail* and *Slug* and of the major mesenchymal marker *Vimentin* in association with the break-down of the epithelial marker *E-cadherin*. These molecular rearrangements are mirrored by cytoskeletal remodeling along with increased cell motility and reduced cell adhesion capability to fibronectin substrate. The assessment of the EMT transdifferentiation highlights that either NAC or AMD3100 or IκB alpha treatment is able to attenuate the increase of *Snail, Slug* and *Vimentin* as well as the migratory and the adhesion abilities of K562 lacking of FtH expression. On the contrary, none of the above mentioned treatments restore *E-cadherin* levels in K562^shFtH^ cells suggesting the non-involvement of ROS/CXCR4/NF-κB molecular axis in the regulation of this marker but rather the existence of other underlying molecular regulatory mechanisms such as the previously reported epigenetic imprinting (69).

To the best of our knowledge this is one of the few reports highlighting the role of ROS in the acquisition of characteristics ascribable to EMT phenotype in cells of hematological origins. Moreover, we describe a link between iron metabolism and CXCR4 in the hematological malignancies which may suggest a potential mechanism through which leukemic cells acquire a metastatic phenotype and a tendency to move to a distal organ. Finally, we believe that iron metabolism might be considered as part of the dynamic crosstalk between hematopoietic cancer cells and their microenvironment and that a perturbation of this crosstalk affects the metastatic potential in the hematological malignancies. Clearly, the cellular context of iron/redox metabolism in the modulation of this phenotype is important; hence, cell- or leukemia-subtype specific dependence of this new molecular axis would be the focus of future studies.

## Data Availability Statement

The raw data supporting the conclusions of this article will be made available by the authors, without undue reservation.

## Author Contributions

RC, IA, FB, and FC conceived and designed the study. RC, IA, AD, ASal, AB, ASac, MD, and FB performed the experiments. RC, IA, MF, BQ, CP, FB, and FC analyzed the data. RC, IA, FB, and FC wrote the first draft of the manuscript. All authors contributed to manuscritp revision, read and approved the submitted version.

## Conflict of Interest

The remaining authors declare that the research was conducted in the absence of any commercial or financial relationships that could be construed as a potential conflict of interest. The handling editor declared a past co-authorship with one of the authors AD.
