# Supplementary material for: FtH-Mediated ROS Dysregulation Promotes CXCL12/CXCR4 Axis Activation and EMT-Like Trans-Differentiation in Erythroleukemia K562 Cells
Source: Front Oncol. 2020 May 5;10:698. doi: 10.3389/fonc.2020.00698 (PMC7214836; doi:10.3389/fonc.2020.00698)
Supplement: Supplementary file 1 [file Image_1.pdf]

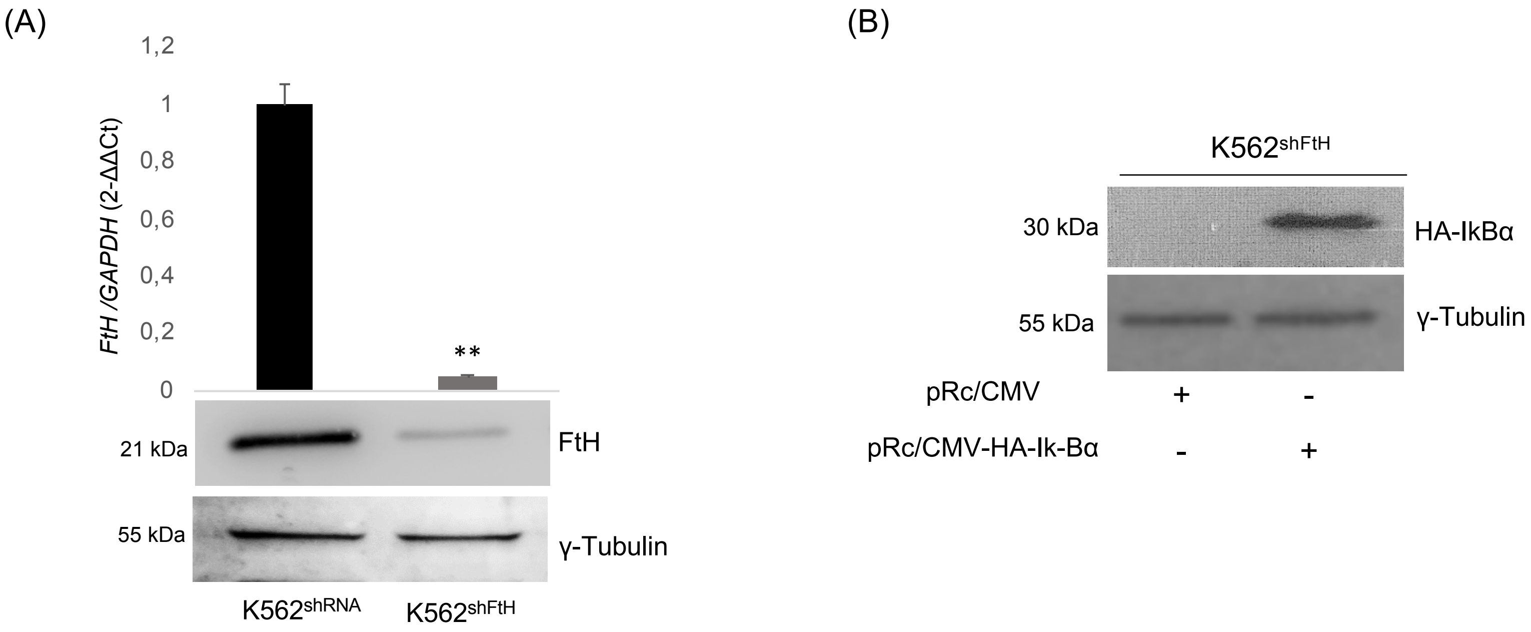

Figure S1. (A) *FtH* silencing. qPCR (up) and Western Blot analysis (down) of FtH in $K562^{shRNA}$ and $K562^{shFtH}$ cells. Final results represent mean ± SD of three independent experiments, **$p<0.01$. γ-Tubulin was used as loading control. **(B)**. Representative Western Blot analysis for HA-IkBα expression in $K562^{shFtH}$ cells. γ-Tubulin was used as loading control.
